# Biotechnological applications of functional metagenomics in the food and pharmaceutical industries

**DOI:** 10.3389/fmicb.2015.00672

**Published:** 2015-06-30

**Authors:** Laura M. Coughlan, Paul D. Cotter, Colin Hill, Avelino Alvarez-Ordóñez

**Affiliations:** ^1^Teagasc Food Research CentreCork, Ireland; ^2^Alimentary Pharmabiotic CentreCork, Ireland; ^3^School of Microbiology, University College CorkCork, Ireland

**Keywords:** functional metagenomics, industrial applications, food, pharmacological, catalysts, bioactives, antimicrobials

## Abstract

Microorganisms are found throughout nature, thriving in a vast range of environmental conditions. The majority of them are unculturable or difficult to culture by traditional methods. Metagenomics enables the study of all microorganisms, regardless of whether they can be cultured or not, through the analysis of genomic data obtained directly from an environmental sample, providing knowledge of the species present, and allowing the extraction of information regarding the functionality of microbial communities in their natural habitat. Function-based screenings, following the cloning and expression of metagenomic DNA in a heterologous host, can be applied to the discovery of novel proteins of industrial interest encoded by the genes of previously inaccessible microorganisms. Functional metagenomics has considerable potential in the food and pharmaceutical industries, where it can, for instance, aid (i) the identification of enzymes with desirable technological properties, capable of catalyzing novel reactions or replacing existing chemically synthesized catalysts which may be difficult or expensive to produce, and able to work under a wide range of environmental conditions encountered in food and pharmaceutical processing cycles including extreme conditions of temperature, pH, osmolarity, etc; (ii) the discovery of novel bioactives including antimicrobials active against microorganisms of concern both in food and medical settings; (iii) the investigation of industrial and societal issues such as antibiotic resistance development. This review article summarizes the state-of-the-art functional metagenomic methods available and discusses the potential of functional metagenomic approaches to mine as yet unexplored environments to discover novel genes with biotechnological application in the food and pharmaceutical industries.

## Introduction

Recent advances in molecular microbiology have revealed that the microbial world extends far beyond what can be revealed by traditional microbiological techniques. Environments once believed to be devoid of life have now been shown to support the growth of microbes. As a consequence, it is now accepted that microorganisms thrive throughout nature, and that at least some microorganisms can be found in almost all known environments. This is due to the fact that microbial life has adjusted to survive under a wide range of harsh or unaccommodating conditions, resulting in a variety of diverse microorganisms adapted to specific niches. This review article explores the molecular methods that can provide access to these specially adapted microbes and, more specifically, their potentially useful genes/molecules and outlines how these approaches can be harnessed by the food and pharmaceutical industries.

Traditional microbiology generally involves obtaining a pure culture as a major step in any study. However, it is estimated that standard laboratory culturing techniques provide information on 1% or less of the bacterial diversity in a given environmental sample (Torsvik et al., 1990). This is most noticeable in what is known as the plate count anomaly, i.e., the discrepancy between the numbers of microorganisms detected by microscopy and the numbers obtained from pure colony counts of cultivated samples (Staley and Konopka, 1985). Although significant advances have been recently made in culturing as-yet-uncultured microbes, e.g., Ling et al. (2015), culture-independent techniques present a more promising effort to access the genetic information contained within the vast number of species in the environment.

Metagenomics presents a molecular tool to study microorganisms *via* the analysis of their DNA acquired directly from an environmental sample, without the requirement to obtain a pure culture. With this technology, the DNA of microorganisms in a population is analyzed as a whole. Sequencing and analysis of total metagenomic DNA can provide information about several aspects of the sample, allowing one to better characterize the microbial life in a given environment. It can not only reveal the identity of species present but also can provide insight into the metabolic activities and functional roles of the microbes present in a given population (Langille et al., 2013). Expression of the genetic information from an environmental sample in a routinely culturable surrogate host can also overcome in part the barriers faced when dealing with as yet uncultured bacteria. The coupling of this approach with function-based screening of the subsequent colonies to uncover a desired activity that has been conferred onto the host by the inserted environmental DNA in a functional metagenomics approach is a powerful technique for the discovery of novel functional genes from uncultured microorganisms.

In this review article, functional metagenomics is discussed as an emerging molecular technique with potential applications in industrial settings. An overview of the current methodological strategies employed for functional metagenomic analysis of microbial populations, with emphasis on the use of phenotypic-based metagenomic screens for the discovery of novel small molecules, enzymes, and bioactives is provided. The applications of such compounds to the food and pharmaceutical industries are discussed, while highlighting recent successes in this area.

## Functional metagenomics: methodological approaches

### Sequencing-based strategies

**Figure 1:**
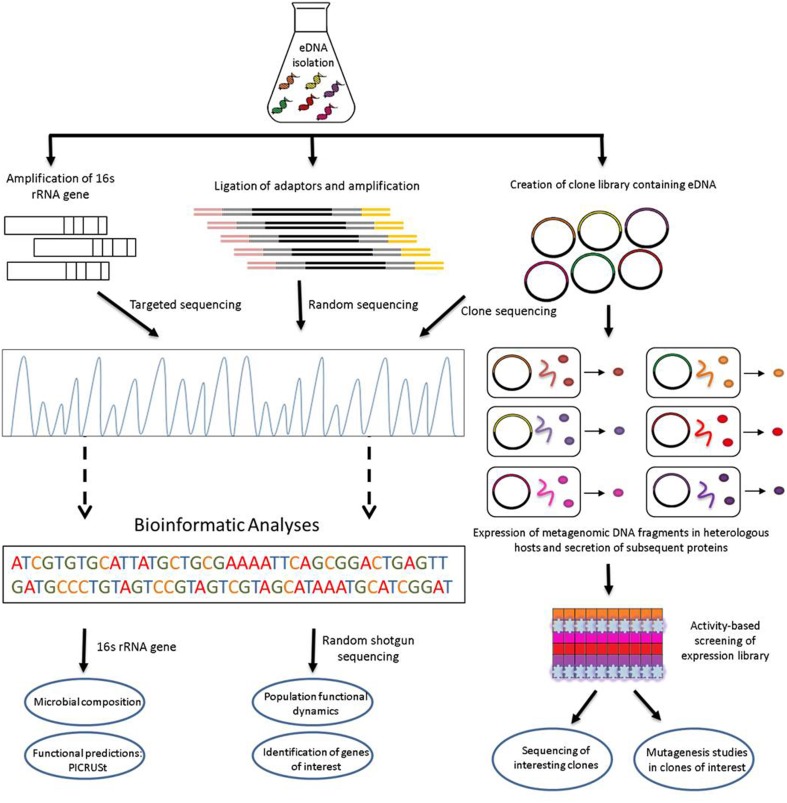
**Schematic view of functional metagenomic strategies for the identification of novel biocatalysts and bioactives from environmental DNA**.

Metagenomic analyses begin with the isolation of microbial DNA from an environmental sample. The acquired metagenomic DNA specimen should be as pure and of as high quality as possible, and should accurately represent all species present both qualitatively and quantitatively. Direct sequencing of extracted metagenomic DNA, followed by appropriate bioinformatics analyses, can facilitate the elucidation of the functional traits of microorganisms colonizing particular environments (Figure 1).

The initial break from culture-dependent to culture-independent approaches for the microbiological analysis of an environmental sample involved the sequencing of genes encoding microbial ribosomal RNAs (rRNAs). Highly conserved primer binding sites within the bacterial 16S rRNA gene facilitate the amplification and sequencing of hypervariable regions that can provide species-specific signature sequences useful for bacterial identification in an environmental sample (Lane et al., 1985). This technology enables microbiologists to determine phylogenetic relationships between unculturable bacteria and assess and quantify the microbial consistency of a sample. In addition, through 16S rRNA gene sequencing of a metagenomic sample, a functional profile of the bacteria present in a given environment can also be obtained. Information regarding the functional roles of already studied bacterial species is available in database archives, including both cultured bacteria whose functional proteins have been extensively characterized as well as functions assigned to bacterial proteins produced by uncultured bacteria through previous metagenomic studies. Once a member of a previously described bacterial family has been identified in an environmental sample, or an appropriate closest known relative has been appointed, phylogenetic analysis may assign predicted functions to an identified bacterial species by referring to the functional information available regarding that particular taxonomic group. This process can be applied to potentially most, if not all, of the different bacterial species encountered in a sample and therefore community roles can be predicted for the microbes dwelling in the sampled niche without the need for shotgun sequencing (described below). Phylogenetic Investigation of Communities by Reconstruction of Unobserved States (PICRUSt) is a computational approach developed by Langille et al. (2013) which can be used to predict the functional properties of microorganisms in a metagenomic sample from characterized relatives in available databases using 16S rRNA sequencing data. By quantifying the individual species abundance in a sample and, in doing so, quantifying the function(s) assigned to that family, PICRUSt can predict the overall functional composition of the community. Keller et al. (2014) also explored this concept through a combinatorial approach of 16S rDNA metabarcoding and single genomics for assessing the compositional and functional diversity of a microbial community. Although these authors were successful in validating their method, this innovative technique requires optimization prior to its introduction into larger and more challenging projects. Microbial eukaryotic communities may also be studied through similar strategies. Eukaryotic-specific primers homologous to the bacterial 16S rRNA can be used to target eukaryotic microbes present in an environmental sample. Bates et al. (2012) used bar-coded pyrosequencing of 18S rRNA to investigate the eukaryotic components of three different lichens, identifying members of the Alveolata, Metazoa, and Rhizaria taxonomic clades. Non-coding DNA located between the small and large subunit eukaryotic rRNA genes, known as the Internal Transcribed Spacer (ITS) regions, are also targeted as a universal DNA marker in Fungi (Schoch et al., 2012). The environmental virome has also been explored through metagenomics by the coupling of sequence-independent amplification of viral nucleic acids with next generation sequencing technologies (Smits and Osterhaus, 2013), particularly in the areas of epidemiology and diagnostics. In addition, genes similar to those of metabolic cells, known as auxiliary metabolic genes (AMGs), have been discovered in viruses (reviewed by Rosario and Breitbart, 2011) and may have potential in the search for industrially relevant enzymes and bioactives.

Environmental DNA random shotgun sequencing, where total metagenomic DNA is sequenced, assembled and annotated, has been shown to be a more useful tool which may be used to analyse at a molecular/species level the metagenome of an environmental sample. In this instance, the functional potential of a microbial population is revealed by directly sequencing the environmental DNA rather than predicting its functional potential based on 16S rRNA data. Some examples of large scale metagenomic studies involving shotgun sequencing are those carried out by Venter et al. (2004), who characterized the microbial population of the Sargasso Sea identifying 1.2 million previously undescribed genes including the first assignment of rhodopsin-like photoreceptors to bacterial species, Warnecke et al. (2007), who analyzed the hindgut paunch microbiota of a *Nasutitermes* species of wood-feeding termite revealing unprecedented diversity of the microbial community and identifying novel genes involved in cellulose and xylan hydrolysis, Oh et al. (2014), who analyzed the microbial content and subsequent functional capacity of the healthy human skin microbiome through shotgun metagenomic sequencing, and Hess et al. (2011), who deep sequenced 268 gigabases of metagenomic DNA obtained from the microbiota of cow rumen unveiling carbohydrate active genes encoding enzymes capable of degrading biomass, a desirable ability in the development of biofuels as a renewable energy source. Random sequencing of shotgun metagenomic DNA may reveal genes of interest, the probable phylogeny of which can be inferred through searches for homology in non-redundant databases, usually via Basic Local Alignment Search Tool (BLAST) analysis. Thus, random sequencing has the potential to identify the presence of already known genes, with reported beneficial functions, or their homologs in an uncultured microorganism, which can provide additional advantages and improve the functionality of in-use proteins/enzymes/catalysts, e.g., the new variant/homolog may encode a protein that is capable of carrying out a specific catalytic or metabolic function and may also be tolerant to an extreme environment habitually encountered in industrial processes. This approach is also useful for the study of the population dynamics of a community, including genomic evolution (Chandler et al., 2014; Kay et al., 2014) and the distribution and redundancy of functions throughout the community (Mendes et al., 2015).

Nevertheless, the sequence-based approaches to analysing environmental samples are limited to the study and identification of genes and DNA sequences homologous to those that are already known. Consequently, the possibility of using sequence-based methods for the discovery of proteins encoded by novel sequences is restricted. Phenotypic-based screening of constructed metagenomic expression libraries, described in the next section of the manuscript, is better suited to the unearthing of previously undescribed proteins and small molecules.

### Phenotypic-based strategies

Functional metagenomic analyses can be carried out on metagenomic libraries *via* the isolation and purification of DNA from an environmental sample, cloning of the DNA into a suitable vector, heterologous expression of the insert vector containing environmental DNA fragments in a suitable surrogate host (usually *Escherichia coli*), and analysis of subsequent transformants by either sequencing- or phenotypic-based approaches, or both (Figure 1). Screening of metagenomic libraries through phenotypic-based approaches is carried out to detect the expression of a particular phenotype conferred on the host by inserted DNA. Screening is usually performed on multiple clones simultaneously on a fixed matrix in which the entire group is assayed with an appropriate indicator to reveal the presence of a phenotypically relevant clone. Such assays require the functional protein to be secreted from the host cell to allow for extracellular detection. Metagenomic clones may be grown on specific indicator media, to allow visual identification of an active clone, e.g., hemolytic activity on blood agar (Rondon et al., 2000), lipolytic activity (Henne et al., 2000), etc. In other occasions, the presence of zones of inhibition in soft agar overlay assays using indicator microorganisms can reveal inhibitory or antimicrobial agents produced by an active clone (Tannieres et al., 2013; Iqbal et al., 2014). Libraries may also be screened based on selection approaches. In these circumstances only the clones onto which the activity of interest has been conferred by the metagenomic DNA insert will grow or survive. Selections include for instance the ability to metabolize a given substrate as a clone's sole carbon source (Entcheva et al., 2001), the ability to resist a potent antimicrobial agent (Donato et al., 2010) or the ability to grow in the presence of a lethal concentration of a heavy metal (Staley et al., 2015).

An alternative option for the identification of novel genes, the Substrate-Induced Gene EXpression screening (SIGEX), was developed by Uchiyama et al. (2005). It relies on the principle that catabolic gene expression is generally induced by a specific substrate or metabolite of catabolic enzymes and is controlled by regulatory elements situated close to these genes. With SIGEX, the environmental DNA inserts are fused with a reporter gene encoding green fluorescent protein (*gfp*) on an operon-trap vector and induced by a target substrate. SIGEX is combined with fluorescent-activated cell sorting (FACS) for the high-throughput selection of GFP-expressing clones. Additionally, the protocol eliminates the incorporation of clones containing self-ligated plasmids and those that are constitutively expressing GFP. Despite some limitations with regard to the applications of this method (reviewed by Yun and Ryu, 2005), SIGEX is an efficient process for the identification of novel catabolic substrate-induced genes. Uchiyama and Miyazaki (2010) went on to expand the capabilities of the SIGEX protocol and developed a reporter assay system for the screening of metagenomic libraries for enzymatic function called Product-Induced Gene EXpression (PIGEX). The system uses a transcriptional activator, which is sensitive to the product of the desired reaction, placed upstream of a *gfp* gene insert. Should a clone possess the activity of interest, upon exposure to an appropriate substrate, the product of this reaction activates transcription of the chosen transcriptional regulator and in turn *gfp* causing the clone to fluoresce, allowing easy detection of positive clones. Pooja et al. (2015) identified through PIGEX a periplasmic α-amylase from a cow dung-derived metagenomic library by isolating an active clone that fluoresced in response to a maltose substrate.

Despite the potential usefulness of such systems, phenotypic-based functional metagenomic approaches face a number of complications, to which potential resolutions are currently being devised. To successfully identify a useful gene or protein candidate a series of sequential steps in the cloning and screening process must occur adequately and effectively. Transcription of the entire gene, translation of its mRNA, correct protein folding, and secretion of the active protein from the surrogate host must all be achieved before functional screening even begins. Suitable and efficient screening methods must also be applied to detect the presence of an interesting gene within the metagenomic library. As the probability of identifying a metagenomic clone, among possibly thousands of others, with a specific desired activity is low (Uchiyama and Miyazaki, 2009), high-throughput screening (HTS) protocols may improve the chances of obtaining an active clone, by allowing higher numbers of clones to be screened simultaneously. An obstacle occurring at any of these stages may result in the overlooking of an interesting clone which might have been detected under the correct circumstances.

One aspect of the methodological approach that can be particularly challenging relates to expressing DNA fragments isolated from microorganisms native to diverse and exotic environments in a relatively domesticated host such as *E. coli* (Banik and Brady, 2010). Even if the foreign DNA is successfully transcribed and translated (perhaps due to the presence of DNA regulatory elements placed on the vector), the correct chaperones required for proper protein folding in the original species may be absent from *E. coli*. A strategy being explored to overcome host related limitations is the generation of an alternative surrogate expression host that may be more suited to efficiently expressing the environmental DNA at hand. Craig et al. (2009) discovered two novel compounds through functional screening of a soil derived metagenomic library expressed in *Ralstonia metallidurans*. The library was constructed using *E. coli* as a heterologous host and then the DNA transferred to *R. metallidurans* for activity based screening. Two clones showed activity in *R. metallidurans*, one displaying antimicrobial activity through the expression of a polyketide synthase gene and a second yellow colored clone expressing a carotenoid gene cluster. Clones active in *R. metallidurans* did not confer the same metabolic abilities onto the *E. coli* host. This shows the importance of using additional heterologous hosts to identify active clones which may not be expressed in the standard *E. coli* host. After their initial success, this research group carried out a study to compare six different Proteobacteria as hosts for the same soil derived metagenomic cosmid library (Craig et al., 2010). Each host expressing the library was functionally screened for antimicrobial activity, pigment production and altered colony morphology conferred onto the host by the DNA insert. Bacterial species from common soil-dwelling phyla were chosen as experimental hosts. Five candidate hosts, *Agrobacterium tumefaciens, Burkholderia graminis, Caulobacter vibrioides*, *Pseudomonas putida*, and *Ralstonia metallidurans*, were compared to the standard and most commonly used host, *E. coli*. Active clones were recovered from the library, having been expressed by different heterologous hosts with minimal overlap between hosts. This study shows the usefulness of Broad-Host Range vectors for overcoming host expression related barriers. Biver et al. (2013a) carried out a study to evaluate the use of an *E. coli-Bacillus subtilis* shuttle vector to functionally screen a forest soil-derived metagenomic library for antimicrobial activity. Activity based screening identified a novel antimicrobial agent, shown to be proteinaceous in nature though not yet fully characterized, that is active against *Bacillus cereus*. The DNA fragment responsible for such activity was active in the *B. subtilis* host alone and no activity was observed when the fragment was expressed in *E. coli*. Again, the importance of developing multiple host expression systems is highlighted by these findings. Further studies similar to those mentioned above must be carried out to better characterize and therefore more fully understand potential alternative hosts. Another obstacle faced in heterologous expression is the possibility of a DNA fragment being too short to contain a functional gene cluster or operon. The availability of a vector able to accommodate large DNA inserts is also fundamental (Streit and Schmitz, 2004). The use of large insert vectors capable of accommodating biosynthetic gene clusters or operons, and the development of shuttle vectors capable of propagating in more than one heterologous host, are examples of strategies being explored to overcome methodological limitations.

## Applications of interest of functional metagenomics in food and pharmaceutical industries

### Discovery of novel bio-catalysts

Certain microbial enzymes are of particular interest to the food and pharmaceutical industries for the catalysis of reactions which may be difficult or expensive to maintain. This interest stems from the fact that there is often difficulty in synthesizing chemical catalysts that truly mimic the complexity of biological enzymes. Many industrial processes are associated with a large environmental burden. Substituting traditional chemical processes used to produce certain compounds or molecules with enzymatic pathways naturally sourced is a more environmentally friendly approach to large-scale production. As microorganisms can catalyze a vast range of reactions, they are an obvious source of enzymes for industrial applications. Several authors have explored this avenue in the last decade (Table 1).

**Table 1:** **Some novel enzymes of industrial interest discovered through functional metagenomics**.

**Enzyme**	**Closest known homolog**	**Method/Host**	**Environment**	**References**
Four lipolytic enzymes	Moderate identity (<50%) to lipolytic proteins from *Streptomyces, Moraxella, Acinetobacter*, and *Sulfolobus* sp.	Activity based screening of *E. coli* plasmid library	Soil from a meadow, a sugar beet field and the Nieme River valley, Germany	Henne et al., 2000
Low pH, thermostable α-amylase	High sequence similarity to α-amylase of *Pyrococcus* sp. KOD1	Function-based screening of *E. coli* plasmid library followed by expression of gene of interest in *Pseudomonas fluorescens* for functional evaluation	Deep sea and acid soil	Richardson et al., 2002
12 esterases, 9 endo-β-1,4-glucanases, and 1 cyclodextrinase	Various putative source organisms	Functional screening of lambda phage library transformed into *E. coli*	Rumen of dairy cow	Ferrer et al., 2005b
Three ß-glucanases	Low sequence identities to known ß-glucanases. Other sequences present in one of the inserts showed identity to *Bacteroides* sp.	Function-based screening of *E. coli* BAC library	Large bowel of mouse	Walter et al., 2005
β-agarase	77% identity to corresponding protein in *Pseudoalteromonas atlantica*	Activity based screening of *E. coli* plasmid library	Soil	Voget et al., 2003
Two esterases	One esterase showed 83% identity to metagenome-derived EstA3 (AAZ48934) and 59% identity to a betalactamase (YP_003266771) of *Haliangium ochraceum* DSM 14365. The other esterase showed 37% identity to a hypothetical protein from *Neisseria elongata*	Activity based screening of two separate libraries: (plasmid and fosmid) transformed into *E. coli*	Soil Water	Ouyang et al., 2013
Two esterases	One esterase showed 51% identity to a class C ß-lactamase from *Burkholderia pseudomallei* and was also 61% similar and 45% identical to a functional esterase (AAF59826) from *Burkholderia gladioli*. Second esterase showed 59% identity to a ß-lactamase from *Sphingopyxis alaskensis*	Activity based screening of two *E. coli* cosmid libraries	Soil Drinking water	Elend et al., 2006
Esterase	Unidentified mesophilic soil microbe	Activity based screening of *E. coli* plasmid library	Environmental soil samples: mudflats, beaches, forests	Kim et al., 2006
Thermostable esterase	64% similarity to an enzyme from *Pyrobaculum calidifontis*	Activity based screening of *E. coli* fosmid library	Mud Sediment-rich water	Rhee et al., 2005
Two esterases	One esterase showed highest identity (64.9%) to a putative esterase (YP_220901) from *Brucella abortus biovar* 1. The other esterase showed highest identity (40.4%) to a putative esterase (ZP_01658665) from *Parvibaculum lavamentivorans*	Activity based screening of *E. coli* BAC library	Surface seawater, South China Sea	Chu et al., 2008
Six lipolytic clones	The six clones individually showed highest identity to the following proteins: (i) Esterase/lipase (ZP_00034241), *Burkholderia fungorum*, (ii) Thermophilic carboxylesterase (1EVQA), *Alicyclobacillus acidocaldarius* (iii) Thermophilic carboxylesterase (1EVQA), *A. acidocaldarius* (iv) Esterase/lipase (ZP_00034303), *B. fungorum* (v) Esterase/lipase (ZP_00034303), *B. fungorum* (vi) Esterase HDE (BAA82510), petroleum-degrading bacterium HD-1	Activity based screening of *E. coli* fosmid library	Forest topsoil	Lee et al., 2004
Cellulase (β-glucosidase activity)	Low sequence identity to *Plasmodium* and *Borrelia* species	Function-based screening of *E. coli* library	Soil	Jiang et al., 2009
Glycosyl hydrolase	>60% identity to β-1-4-endoglucanase from *Prevotella bryantii* B14 (AAC97596) and β-1-4-xylanase from *Prevotella ruminicola* 23 (AAC36862). 100% identity to a partial sequence (AAB20175) of the N terminus B14 enzyme from *P. bryantii*.	Functional screening of lambda phage library transformed into *E. coli*	Cow rumen fluid	Palackal et al., 2007
137 nitrilase genes (Relevant in fine chemical synthesis in drug manufacture)	Varying degrees of amino acid sequence similarity to proteins from several sequence clades within the nitrilase subfamily	A phagemid library expressed in *E. coli* screened by selection for the ability to grow on a nitrile substrate	Soil Water	Robertson et al., 2004
Halotolerant and moderately thermostable tannase	New member of tannase superfamily	Activity-based screening of *E. coli* plasmid library	Cotton field soil	Yao et al., 2011
Three carboxylic ester hydrolases	77% amino acid identity to lipolytic enzyme (AEM45126) from German forest soil-derived metagenomic library	Activity-based screening of *E. coli* plasmid library	Forest soil	Biver and Vandenbol, 2013
Alkaline serine protease	Most closely related to an alkaline protease isolated from *Bacillus* sp.	Activity-based screening of IPTG-inducible vector library expressed in *E. coli*	Forest soil	Biver et al., 2013a
Fibrinolytic metalloprotease (zinc-dependent)	Amino acid sequence showed 46% identity to metallopeptidase from *Dechloromonas aromatica* (AAZ45577)	Activity-based screening of *E. coli* fosmid library	Mud, Korean west coast	Lee et al., 2007
Two serine proteases	First novel protease: 52% amino acid identity to a thermophilic alkaline protease from *Geobacillus stearothermophillus* (AAK29176). Second novel protease: 51% sequence identity with a putative protease of *Bacillus sphaericus* (CAB46075)	Activity-based screening of *E. coli* plasmid and fosmid libraries	Surface sand from Gobi and Death Valley deserts	Neveu et al., 2011
Alkaline serine protease	98% sequence similarity with uncharacterized proteases of various *Shewanella* sp.	Activity-based screening of *E. coli* plasmid library	Goat skin surface	Pushpam et al., 2011
Cold-active lipase	91% identity to a known lipase from *Pseudomonas fluorescens* B68 (AY694785)	Activity based screening of *E. coli* cosmid library	Oil-contaminated soil, Northern Germany	Elend et al., 2007
Moderately thermostable (and thermally activated) lipase	*Acidobacteria* phylum	Activity based screening of *E. coli* fosmid library	Soil, Brazilian Atlantic Forest	Faoro et al., 2012
Five esterases	Two did not show significant sequence identity to known esterases, the remaining genes showed low to moderate identity to known esterases	Activity based screening of *E. coli* phagemid vector library	Brine: seawater interface, Uranian hypersaline basin	Ferrer et al., 2005a
Thermostable family VII esterase with high stability in organic solvents	45% identity to *Haliangium ochraceum* DSM 14365 (ACY17267)	Activity based screening of *E. coli* fosmid library	Compost	Kang et al., 2011
Alkaline-stable family IV lipase	83% identity with a cold-active esterase from a deep-sea metagenomic library (ADA70028). 59% identity with an esterase from *Vibrio splendidus* LGP32 (YP_002394831)	Activity based screening of *E. coli* plasmid library	Marine sediment, South China Sea	Peng et al., 2014
Protease-insensitive feruloyl esterase	56% identity to predicted esterase from *Eubacterium siraeum* V10Sc8a (CBL34630). 55% identity to predicted esterase from *E. siraeum* (CBK96609)	Function-based screening of *E. coli* fosmid library	China Holstein cow rumen	Cheng et al., 2012a
Xylanase	44% identity to glycoside hydrolase family protein from *Clostridium thermocellum* ATCC 27405 (YP001038252)	Function-based screening of *E. coli* fosmid library	China Holstein cow rumen	Cheng et al., 2012b
Two UDP glycotransferase (UGT) genes. One is a novel macroside glycotransferase (MGT)	The first one is weakly similar (71% similarity) to hypothetical UGT from *Fibrisoma limi*. The second one is highly similar to a hypothetical MGT from *Bacillus thuringiensis*	Thin layer chromatography (TLC)-based functional screening of *E. coli* fosmid library	Elephant feces, Hagenbeck Zoo, Germany. Tidal flat sediment, Elbe river, Germany.	Rabausch et al., 2013
Cold-adapted ß-galactosidase	Highest percentage identities to β-galactosidases from *Planococcus* sp. “SOS Orange” (39%), *Planococcus* sp. L4 (39%), and *Bacillus halodurans* C-125 (39%)	Function-based screening of *E. coli* plasmid library followed by expression of gene of interest in *Pichia pastoris* for analysis and characterization	Topsoil samples, Daqing oil field, Heilongjiang Province in China	Wang et al., 2010
Cold-active ß-galactosidase	53% identity to β-galactosidases from *Clostridium hathewayi*	Function-based screening of *E. coli* plasmid library.	Ikaite columns SW Greenland	Vester et al., 2014
ß-galactosidase	Not available	Function-based screening of *E. coli* plasmid library followed by expression of gene of interest in *Pichia pastoris* for functional evaluation	Not available	Wang et al., 2012
11 amidase genes (Three novel)	Three novel amidases: the first showed highest identity (54%) to putative isochorismatase hydrolase from *Streptomyces* sp. strain AA4; the second showed 45% primary amino acid sequence identity with a hypothetical protein (further information not available); the third showed 57% primary amino acid sequence identity with a protein that contains a transmembrane ABC transporter signature motif and possibly encodes a polypeptide with amidase activity	PIGEX-based screening of benzoate-responsive sensor plasmid library transformed into *E. coli*	Activated sludge from aeration tank of a coke plant; wastewater treatment plant, Japan	Uchiyama and Miyazaki, 2010
Periplasmic α-amylase	100% similarity with *mal*S gene in *E. coli* (X58994.1)	PIGEX-based screening of maltose-induced plasmid library transformed into *E. coli*	Cow dung, India	Pooja et al., 2015
37 genes with lipolytic activity	29–90% sequence identity to known and putative proteins from numerous different species, including uncultured bacteria	Activity based screening of *E. coli* plasmid and fosmid libraries	Forest soil, Germany	Nacke et al., 2011

Novel enzymes from natural sources are extremely useful in food processing reactions. Many of these relate to reactions that occur in nature to process food for energy but are difficult to mimic on an industrial level, e.g., degradation of starch. In other instances, the search has focused on enzymes that can carry out reactions under extreme conditions, which often prevail in food processing, e.g., high temperatures and extremes of pH. Indeed, microbial enzymes are used for brewing, baking, synthesis of sugar and corn syrups, starch and food processing, texture and flavoring, processing of fruit juices, and production of dairy products and fermented foods, among others, either as recombinant enzymes or by using starter cultures with desirable activities. The following are some examples of industrial food processes which have benefited (and may continue to do so) from access to the diverse repository of enzymes possessed by microorganisms.

In the food industry, starch harvested from sources such as maize, wheat, and potatoes is processed to yield food products such as glucose and fructose syrups, starch hydrolysates, maltodextrins, and cyclodextrins (reviewed by van der Maarel et al., 2002). In recent times, the chemical hydrolysis of starch, which involves acid treatment, is being replaced with enzymatic digestion by starch-hydrolyzing enzymes obtained from natural sources. Starch-modifying enzymes are also added to dough in the baking industry to act as bread anti-staling agents. These starch-converting enzymes usually originate from the α-amylase family or family 13 glycoside hydrolase. Amylases from microbial sources are used in starch processing such as α-amylases from *Geobacillus stearothermophilus* and *Bacillus licheniformis*. However, despite the advantages of using enzymatic over chemical hydrolysis (high specificity of enzymes, milder reaction conditions, natural means of processing more acceptable to consumers and to the public), there are limitations with the enzymes currently being used. Starch hydrolysis is carried out at high temperatures, at which α-amylases are usually not active at a pH below 5.9. For the reaction to proceed efficiently, the pH must be raised by the addition of NaOH. As these enzymes also exhibit a Ca^2+^ dependency, Ca^2+^ must be added to the reaction in addition to adjusting the pH. Thermostable, Ca^2+^-independent α-amylases with low pH activity would be ideal for the starch hydrolyzing process. Richardson et al. (2002) identified an α-amylase optimal for the corn wet milling process. They carried out activity based screenings under conditions of temperature and pH similar to those of the corn wet milling process on a large library of metagenomic clones constructed from diverse environmental samples. The clones were also phylogenetically screened for homology to known α-amylases. Three clones were selected which performed well under the given conditions. Phylogenetic analysis revealed that all three enzymes were members of the glycosyl hydrolase family 13. They were expressed in *Pseudomonas fluorescens* and their activity was compared to the enzymes currently used in industry (from *B. licheniformis*). One clone was found to have better characteristics for application to the corn wet milling process than the enzyme currently in use. However, further research is needed to improve the low yield of enzyme produced under industrial conditions.

Lipases and esterases are hydrolytic enzymes which play important roles in the food and pharmaceutical industries. Lipases hydrolyze fats into fatty acids and glycerol at the water lipid interface and reverse the reaction in the non-aqueous phase (Gupta et al., 2004). Lipases are exploited by the dairy industry for the hydrolysis of milk fat, releasing short-chain and long-chain fatty acids, creating such features as richness, creaminess or cheesiness depending on the degree of lipolysis, as reviewed by Hasan et al. (2006). For this reason, it is important to use the correct lipolytic enzyme to achieve the right flavor in the final product. Peng et al. (2014) screened a metagenomic library constructed from a Chinese marine sediment for clones displaying lipolytic activity in an *E. coli* host. They discovered a novel highly alkaline-stable lipase with high specificity for butter milkfat esters. Treatment of butter with the newly identified lipase produced rich and distinctive flavors through the production of palmitic and myristic acids while maintaining the cheesy flavor of the short-chain fatty acids. As palmitic and myristic acids are added to food for their distinctive flavor, the hydrolysis of palmitate and myristate in the production of lipolysed milkfat (LMF) to flavor dairy products is a safe and economically viable potential application of the novel lipase identified in this study. Other dairy applications of lipases include the acceleration of cheese ripening and the enhancement of cheese flavor through the synthesis of short chain fatty acids (SCFAs) and alcohols. Lipases are also used in vegetable oil modification and preservation of baked goods (Hasan et al., 2006). Although in the past lipases used in the food industry were predominantly obtained from animal sources, the microbial world potentially holds a wide range of diverse lipases that can be used in many different industrial applications (Table 1). Examples of pharmaceutical applications of lipases sourced from microbes include the synthesis of an intermediate for the production of an anti-tumor agent (Zhu and Panek, 2001) and the synthesis of intermediates of antimicrobial agents (Kato et al., 1997). Also, through the screening of a metagenomic library constructed from an oil-contaminated German soil sample, Elend et al. (2007) identified a lipolytic cold-activated clone which showed high selectivity for esters of primary alcohols and (*R*) enantiomers of non-steroidal anti-inflammatory drugs such as ibuprofen. This enzyme has potential in the pharmaceutical industry for the conversion of such anti-inflammatories into an optically pure form.

Esterases catalyze the hydrolysis of an ester into its alcohol and an acid in aqueous solution. They are distinguished from lipases in that they hydrolyze short-chain over long-chain acylglycerols. In the food industry, esterases are used in fat and oil modification and in the fruit juices and alcoholic beverages industries to produce certain flavors and fragrances, as reviewed by Panda and Gowrishankar (2005). Feruloyl esterases hydrolyze the ester bond between ferulic acid (FA) and polysaccharides present in plant cell wall material. They have a dual usefulness as they not only break down plant biomass (which is useful in industrial waste management) but, in doing so, they de-esterify dietary fibers releasing bioactives with potential beneficial health effects (reviewed by Faulds, 2010). In a study carried out by Cheng et al. (2012a), a metagenomic library constructed from the microbial content of a Chinese Holstein cow rumen was functionally screened for feruloyl esterase activity, identifying a protease-insensitive feruloyl esterase capable of releasing FA from wheat straw. This novel enzyme is of particular industrial interest as it showed high thermal and pH stability and was resistant to several proteases including pepsin. A novel xylanase was isolated from the same metagenomic library (Cheng et al., 2012b) and its ability to work synergistically with the newly discovered feruloyl esterase to release xylooligosaccharides (XOS) and FA from wheat straw was assessed. XOS display prebiotic and gut modulatory activities and have other bioactive properties giving them value as food additives, as reviewed by Moure et al. (2006). The novel xylanase was not only effective in working with the feruloyl esterase, but additionally was capable of improving release of FA from wheat straw at a high dose. Esterases also play a role in the synthesis of chiral drugs including medications to relieve pain and reduce inflammation (Bornscheuer, 2002; Shen et al., 2002; Panda and Gowrishankar, 2005).

ß-galactosidases are widely used in the dairy industry for the hydrolysis of lactose to glucose and galactose. Lactose content in milk is reduced to improve taste (lactose is known to absorb undesirable flavors and odors), to accelerate the ripening of cheeses made from treated milk and for the removal of lactose for the production of lactose-free products for intolerant consumers (reviewed by Panesar et al., 2010). The currently commercially available ß-galactosidase for use in the dairy industry, from *Kluyveromyces lactis*, has a temperature optimum of 50°C and loses much of its enzymatic activity at temperatures below 20°C. Carrying out industrial reactions at lower temperatures is beneficial as it saves energy (and in turn is more economical), it prevents heat destruction of thermosensitive substances such as food compounds, molecules responsible for flavors, taste and nutritional value, etc., and it reduces contamination risks. Cold-active enzymes work at low temperature and can be easily inactivated by rising the temperature to a moderate condition. From a metagenomic library constructed from the ikaite columns of SW Greenland, Vester et al. (2014) isolated a cold-activated ß-galactosidase which can potentially be applied by the dairy industry. The discovered enzyme has an optimal pH of 6 (the natural pH of milk being pH 6.7–6.8) and a temperature optimum of 37°C, but retains lactose hydrolytic activity at 5°C. These properties make it a good candidate for the hydrolysis of lactose into glucose and galactose in milk for the removal of lactose for production of lactose-free products for lactose-intolerant people. In a similar study by Wang et al. (2010) a cold-adapted ß-galactosidase was identified from a metagenomic library expressed in *E. coli*. The insert from the active clone (encoding a full-length ß-galactosidase) was expressed in *Pichia pastoris* to assess its candidacy for use in milk treatment and optimal activity was observed at a temperature of 38°C. The enzyme was active at the natural pH of milk.

Flavonoids are plant secondary metabolites found in numerous dietary fruits and vegetables and whose consumption is beneficial to human health (Ververidis et al., 2007a,b). Flavonoids are difficult to source as they are produced by plants at very low levels. Due to their structural complexity enzymatic modification is preferred over a chemical approach for industrial production. Glycosylation of flavonoids influences their water solubility and bioavailability, making glycosyltransferases that are active on flavonoids of great interest to the food and pharmaceutical industries. Rabausch et al. (2013) developed a novel thin-layer chromatography (TLC) based screening method for the identification of flavonoid-modifying enzymes from a metagenomic library. Two novel flavonoid-modifying enzymes with high activity on flavones, flavonols, flavanones, isoflavones, and stilbenes were discovered in this manner.

Proteases hydrolyze peptide bonds and therefore catalyze the degradation of proteins. They have numerous uses in the food industry, including the tenderizing of meat (Ashie et al., 2002), the coagulation of milk and flavor development in the dairy industry (Huang et al., 2011) and the proteolysis of gluten to achieve gluten-free products in the baking industry (Hamada et al., 2013). Proteases may also be used to release beneficial bioactive peptides from polypeptide chains in certain foods (Hafeez et al., 2014; Mora et al., 2014). Currently, commercial proteases used in the food industry are generally sourced from plants and culturable microorganisms. Proteases from as yet uncultured microbial extremophiles would be of use in the carrying out of proteolysis under unconventional reaction conditions. There have been several novel proteases discovered through functional metagenomic methods. For instance, Biver et al. (2013a) identified an oxidant-stable alkaline serine protease from a forest-soil metagenomic library. An alkaline serine protease was also identified in a metagenomic library constructed from goat skin surface samples by Pushpam et al. (2011). These alkaline proteases are examples of microbial enzymes with potential industrial applications, mainly in the detergent industry.

Tannins are naturally occurring water soluble polyphenols which constitute a large percentage of plant material. Tannases catalyze the hydrolysis of tannins, releasing gallic acid, and glucose. Tannases are used in the food industry as a clarifying agent in the manufacture of beverages such as instant teas, fruit juices, beer, and certain wines (Cantarelli et al., 1989; Boadi and Neufeld, 2001). Tannases are also important to the pharmaceutical industry for catalyzing the release of gallic acid (Sariozlu and Kivanc, 2009) which is used in the production of some antibacterial drugs. Additionally, gallic acid is used in the synthesis of propyl gallate, an antioxidant food additive. Tannases isolated from bacteria have typically been restricted to culturable strains, overlooking the diverse potential of those as yet uncultured. Yao et al. (2011) expressed a metagenomic clone library constructed from cotton field in *E. coli* and screened the transformants for tannase activity, revealing one active clone. Sequence analysis revealed that the active clone encoded a full length tannase gene, which was not found to be closely related to any currently known tannases. Analysis of tannase activity of the enzyme under various industrially relevant conditions was performed and a moderate thermostability of the identified enzyme, which may be useful for food industrial applications, was shown. The enzyme was also found to have a wide range of substrate specificity, making it suitable for applications in both the food and pharmaceutical industries. In 2014, this novel tannase was investigated by Yao et al. (2014) for its suitability for the removal of tannins from a green tea infusion. The presence of tannins in beverages such as green tea is problematic as the ability of tannins to precipitate proteins leads to the formation of a protein haze that is undesirable in terms of product taste and appearance (Wu and Bird, 2010). The tannase enzyme was recombinantly expressed in *E. coli* and immobilized to several matrices, identifying Ca-alginate beads as the most appropriate support. The immobilized enzyme was effective in the removal of tannins from green tea infusion and was found to possess properties distinct from those of the free enzyme, such as high operational and storage stabilities and a higher temperature and pH optimum.

### Discovery of novel bioactives

As with the food industry, the use of microbial enzymes is of particular interest for the biosynthesis of pharmaceutical products previously synthesized *via* chemical means. Thus, functional metagenomics can be applied to the discovery of genes capable of carrying out reactions of interest for the obtaining of bioactives or the synthesis of intermediate compounds in the pharmaceutical industry. One avenue of interest has been the identification and heterologous expression of a microbial biosynthetic pathway capable of producing biotin for industrial purposes (Entcheva et al., 2001; Streit and Entcheva, 2003). Biotin (Vitamin H) is a human and animal dietary requirement and is currently chemically synthesized through industrial processes for addition to food and feed products, with associated negative environmental impacts. The use of biotin-producing microorganisms in place of chemical synthesis offers a greener alternative to conscientious industries. Other microbial biosynthetic genes of interest to the pharmaceutical industry capable of synthesizing other bioactives important for human health and medicine have been also identified by functional metagenomic strategies (listed in Table 2).

**Table 2:** **Some novel bioactives and biosynthetic pathways of industrial interest discovered through functional metagenomics**.

**Bioactive /Pathway**	**Closest known homolog**	**Method/Host**	**Environment**	**References**
Pederin	>80% identity to sequences from *P. aeruginosa*. The discovered *ped* gene cluster is believed to be from a symbiont of the *Paederus* beetle from the genus *Pseudomonas*	Targeted sequencing-based strategy	*Paederus* beetles	Piel, 2002
Biotin	Highest identity to proteins from *Erwinia herbicola*. Significant identity also shown to proteins from *E. coli* and *Pseudomonas putida*	Selelction-based screening of enriched cosmid library in *E. coli* biotin auxotrophic strain	Horse excrement	Entcheva et al., 2001
Known siderophore: vibrioferrin	98% identity to proteins from *Vibrio parahaemolyticus* and *Vibrio alginolyticus*	Function-based screening of *E. coli* plasmid library	Tidal-flat sediment, Ariake Sea	Fujita et al., 2011
Polyketide synthase (PKS) gene	55–59% identity to hypothetical PKS from *Mycobacterium avium* (NP_961164)	Targeted sequencing-based strategy	Marine sponge *Discodermia dissoluta*, Netherlands Antilles	Schirmer et al., 2005
Novel serine protease inhibitor (serpin) gene	Moderate identities to serpins from *Salinibacter ruber* M8 and *Spirosoma linguale* DSM 74. Similarities with possible partial serpins from *Dyadobacter fermentans* DSM 18053, *Arthrospira maxima* CS-328 and *Cyanothece* sp. PCC 7822	Sequence-based screening of *E. coli* plasmid library.	Uncultured marine organisms	Jiang et al., 2011
Borregomycin A and B encoded by *bor* pathway (antiproliferative and antibiotic properties)	ORFs showing 32–86% identity to species from the following genera: *Micromonospora*, *Streptomyces*, *Actinoplanes*, *Corallococcus*, *Cellulomonas*, *Actinomadura*, *Salinispora*, *Microlunatus*, *Modestobacter*, *Frankia*, *Saccharomonospora*, *Nocardia*, *Phaeosphaeria*	Homology guided screening	Soil, Anza-Borrego Desert (CA)	Chang and Brady, 2013
Hypothetical protein with NF-kB pathway stimulatory activity	42% of predicted genes coverage to *B. vulgatus* ATCC 8482	Activity-based screening using a reporter cell line of an *E. coli* fosmid library	Human gut microbiota of Crohn's Disease patients	Lakhdari et al., 2010
Novel prebiotic degradation pathways (11 contigs)	Sequence homology to species of *Bifidobacterium*, *Eubacterium*, *Streptococcus*, *Bacteroides*, *Faecalibacterium*	Hydrolytic activity-based selective screening of two *E. coli* fosmid libraries	Human ileum mucosa and fecal microbiota samples	Cecchini et al., 2013
Five novel putative salt tolerance genes	Identity to hypothetical proteins from genus *Collinsella*, *Eggerthella*, and *Akkermansia*	Function-based screening of *E. coli* plasmid library	Human gut microbiota	Culligan et al., 2012
Novel salt tolerance gene	Not homologous to any sequence at time of study, highest BLAST score to hypothetical protein from *Caulobacter crescentus*	Function-based screening of *E. coli* plasmid library	Faecal sample, healthy 26 year old Caucasian male	Culligan et al., 2013
15 acid resistance genes	37–90% identity to proteins and hypothetical proteins from the following genera: *Thermosinus*, *Streptomyces*, *Candidatus*, *Hyphomicrobium*, *Methylococcus*, *Acidithiobacillus*, *Thioalkalivibrio*, *Nitrosococcus*, *Halorhodospira*, *Haliangium*, *Clostridium*, *Roseomonas*, *Acidiphilium*, *Gemmata*, *Terriglobus*, *Burkholderia*	Function-based screening of six *E*. coli plasmid libraries. Followed by expression in *Pseudomonas putida* and *Bacillus subtilis*	Planktonic and rhizosphere microbial communities of the Tinto River. Five libraries from *Erica andevalensis*, one from headwaters of Tinto River	Guazzaroni et al., 2013

Walter et al. (2005) applied a functional metagenomic method to screen for lichenin-degrading activity in a Bacterial Artificial Chromosome (BAC) library constructed from bacteria obtained from the large-bowel microbiota of mice, identifying three clones with ß-glucanase activity. Glucans cannot be broken down by humans or monogastric animals and so, their hydrolysis relies on bacterial fermentation. As the consumption of glucans is associated with health benefits in humans (Abumweis et al., 2010), glucan hydrolyzing enzymes isolated from bowel-dwelling microbiota may be of interest to pharmaceutical and functional food related industrials. The feed industry may also benefit from the availability of ß-glucanases that improve the digestion of barley-based feed diets by poultry livestock (Von Wettstein et al., 2000).

The development of novel therapeutic strategies relies heavily on gaining a better understanding of human commensals and host-microbe relationships. Lakhdari et al. (2010) established and validated a reporter system capable of detecting immune modulatory activity of metagenomic clones. A metagenomic library constructed from human fecal microbiota of Crohn's Disease (CD) patients was screened for NFkB modulatory activity (whether stimulatory or inhibitory) using an intestinal epithelial cell line transfected with a reporter gene. A clone displaying stimulatory activity of the NF-kB pathway was identified. Although the molecule responsible for the activity is not yet known, two potential candidate loci were determined through transposon mutagenesis: an efflux ABC type transport system and a putative lipoprotein. Phylogenetic analysis showed *Bacteroides vulgatus* to be the closest known homolog to the source of the insert of interest, an interesting finding as *B. vulgatus* is a human gut microbe found to be higher in abundance in CD patients than in a control population. This study presents the development of an innovative platform for screening metagenomic libraries and is likely to inspire the creation of other cell-based screening platforms from which a better understanding of human-microbe symbiotic communications can be obtained, advancing the development of novel therapeutic strategies promoting a healthy relationship with the gut microbiota and in turn the entire human microbiome.

Maintaining gut microbiota homeostasis has been shown to contribute to the overall sustaining of human gut health. Probiotics are an oral infusion of high numbers of live beneficial gut microbes formulated into various yogurts and dairy beverage products that, when ingested in adequate amounts, confer a health benefit on the host (Joint, 2001). As an oral formulation, these products face difficulties in efficacy due to insufficient cell numbers reaching the intestine, owing to the necessity of passing through the majority of the GI tract to reach their site of action in the bowel. The harsh pH and osmolarity of the upper GI tract can destroy a large proportion of the ingested cells. Novel acid and salt resistance mechanisms discovered through functional metagenomic studies similar to those of Guazzaroni et al. (2013), who identified an acid resistant metagenomic clone from the Tinto River environment, and Culligan et al. (2013), who discovered a gene conferring salt tolerance onto an *E. coli* host from a library derived from the human gut microbiota, may be of use in conferring stress resistance to probiotic products. However, this objective faces additional social challenges with respect to consumer acceptance of the use of genetically modified (GM) microorganisms to enhance food products. Although it is generally appreciated by the public that GM cells, organisms and microorganisms are necessary for the production of certain critical biologically active drugs, the thought of everyday food products having been prepared using GM materials is met with a sense of unease, especially in many EU member states. Thus, strict regulations involving the consumption of GM foods and the use of GM organisms in food production and processing have not been made more lenient, as they have in other countries, such as the USA, in recent years. Public transparency and an understanding of the extensive safety and efficacy testing of GM related food products may eventually lead to a change in consumer attitude to bioengineered goods.

Another avenue to maintain human gut health is to promote the growth of beneficial bacteria already present in one's lower GI tract through the use of prebiotics. Prebiotics are non-digestible oligosaccharides (NGOs), usually present in plant material, that are resistant to human digestion in the upper GI tract and are hydrolyzed in the gut by beneficial microbiota to produce SCFAs and organic acids that provide nutritional value to the human host (Gibson and Roberfroid, 1995). Cecchini et al. (2013) used a functional metagenomics approach to investigate the prebiotic hydrolyzing potential of the human gut microbiome by searching for novel prebiotic degradation pathways in a human ileum mucosa and a fecal microbiota derived metagenomic library. They identified high numbers of unknown gut microorganisms capable of hydrolyzing established prebiotics, indicating that the prebiotics tested are not specifically metabolized by their target microorganisms alone. Further investigations must be carried out to determine the effect (if any) of non-specific hydrolysis of prebiotic preparations in the human gut. Galacto-oligosaccharides (GOS) with prebiotic properties can be synthesized through the transgalactosylation activity of ß-galactosidase enzymes on lactose. Wang et al. (2012) validated the ability of a novel ß-galactosidase isolated from a metagenome-derived library for its ability to produce GOS. Carrying out the reaction in an organic-aqueous biphasic media was shown to improve GOS yield. The ß-galactosidase gene discovered in this study is a promising candidate for industrial production of GOS to be used as an additive in various food and dairy products. All of these studies highlight the flexibility of functional metagenomics as a molecular tool not only for identifying new metabolic pathways for biosynthesis of useful/industrially relevant compounds but also for evaluating the efficiency of current therapeutic strategies.

### Discovery of novel antimicrobials

A major driving force behind the biotechnological applications of functional metagenomics is the search for novel antimicrobials effective in medical settings. Microorganisms produce antibiotic molecules to alleviate competitors in their natural habitat. Natural sources have proved fruitful in the past for providing antibiotic molecules, from the discovery of penicillin produced by *Penicillium rubens* in 1928 to date. Although most bacterial infections in humans are curable with current antibiotic therapies, the emergent problem of antimicrobial resistance has led to the prevalence of persistent untreatable infections caused by certain pathogens which have developed a resistance to the used antimicrobial therapy. Antibiotic resistance has challenged medical practitioners and researchers and has led to outbreaks of serious untreatable bacterial infections in clinical settings and even community outbreaks have occurred (Alanis, 2005), making antimicrobial resistance a serious threat to human health (World Health Organization, 2014). The rate of antimicrobial drug discovery has declined in recent years, owing in part to a low drug approval rate by governing bodies (Cooper and Shlaes, 2011) and lesser rewards for manufacturers (Fischbach and Walsh, 2009). The exhaustion of products from culturable microorganisms and preferred use of chemical libraries of pure synthetic compounds over natural product exploration (Li and Vederas, 2009) have also contributed. New advances in metagenomics, high throughput screenings (HTS) and metabolic engineering, e.g., Jayasuriya et al. (2007), provide a new lease of life for natural product drug discovery. Functional metagenomic screens can be applied to the identification of novel antimicrobial molecules by screening microbial populations for antimicrobial activity against indicator or clinically relevant microorganisms. So far, this approach has led to the discovery of several novel antimicrobial compounds (Table 3). Gillespie et al. (2002) described the discovery of two novel antimicrobials (turbomycin A and B) exhibiting broad-spectrum activity against both gram-positive and gram-negative bacteria. These antibiotics were identified through activity-based screening of a metagenomic library from soil samples expressed in an *E. coli* host. Several metagenomic *E. coli* clones expressing antimicrobial activity were discovered by Macneil et al. (2001) through function-based screening of a BAC library constructed from soil microbial DNA. Metagenomic inserts from active clones were found to be related to the compound indirubin, a cyclin-dependent kinases (CDK) inhibitor used in the treatment of human chronic myelocytic leukemia (Hoessel et al., 1999; Marko et al., 2001). An indirubin compound with antimicrobial activity was also identified through activity-based screening of a forest soil metagenomic library by Lim et al. (2005). More recently, Scanlon et al. (2014) developed a HTS method which enabled them to co-culture recombinant clones from a native staphylococcal-derived metagenomic library with the bacterial pathogen *Staphylococcus aureus* in hydrogen-in-oil emulsions, with antibiotic activity being rapidly detected using a fluorescent viability assay. Six clones expressing a lysostaphin gene from *Staphylococcus simulans* with activity against *S. aureus* were identified in this way. Iqbal et al. (2014) constructed a metagenomic library from Arizona soil hosted by *Ralstonia metallidurans*. Functional screening for antimicrobial activity against *Bacillus subtilis* identified six positive clones encoding proteases, a lipase, and enzymes with cell wall lytic activity. These studies highlight the success of applying functional metagenomics to the discovery of novel natural antimicrobials with potential value to the pharmaceutical industry.

**Table 3:** **Some novel antimicrobials, anti-infectives and antimicrobial resistance genes discovered through functional metagenomics**.

**Antimicrobial**	**Closest known relationship (percentage homology)**	**Method/Host**	**Environment**	**References**
Long-chain *N*-acyltyrosine synthase genes	No identity to bacteria cultured at that time. Some similarity to predicted proteins from *Nitrosomonas europaea*, *Desulfovibrio vulgaris*, and *D. desulfuricans*	Activity-based screening of *E. coli* cosmid library	Seven soil samples, Ithaca, NY Boston, MA Costa Rica	Brady et al., 2004
*N*-acyl amino acid biosynthesis gene	Highest similarity to hypothetical protein (MJ1207) from *Methanococcus jannaschii*	Activity-based screening of *E. coli* cosmid library	Soil	Brady and Clardy, 2000
Two isocyanide biosynthetic genes encoding isocyanide-containing antibiotic	Not available. Some identity to known and predicted proteins	Activity -based screening of *E. coli* cosmid library	Soil, Boston, MA	Brady and Clardy, 2005
Violacein biosynthetic gene cluster	Moderate identity to *Chromobacterium violaceum*	Activity -based screening of *E. coli* cosmid library	Soil, Ithaca, NY	Brady et al., 2001
Two ORFs within a clone encoding a transcriptional regulatory protein and a putative indole oxygenase	The indole oxygenase-like protein showed high identity to naphthocyclinone hydroxylase (NcnH) from *Streptomyces arenae*	Activity -based screening of *E. coli* fosmid library	Forest topsoil, Jindong Valley, Korea	Lim et al., 2005
Turbomycin A, B	The ORFs encoding the turbomycins A and B show 53% identity to legiolysin from *Legionella pneumophila*, 54% identity to hemolysin from *Vibrio vulnificus*, 49% identity to 4-hydroxyphenylpyruvate dioxygenase from *Pseudomonas* and 45% identity to MelA in *Shewanella colwelliana*	Activity-based screening of *E. coli* plasmid library	Soil	Gillespie et al., 2002
Uncharacterized protein with antimicrobial activity	Low to moderate sequence identity (26–58%) to proteins and hypothetical proteins from *Solitalea canadensis* DSM 3403 (38 and 46%), *Flavobacterium* sp. CF136 (26%), *Indibacter alkaliphilus* LW1 (40%), *Helicobacter bizzozeronii* CIII-1 (31%) and *Acidovorax* sp. JS42 (58%)	Activity-based screening of *E. coli-Bacillus subtilis* shuttle vector library	Soil sample from a deciduous forest, Belgium	Biver et al., 2013b
Novel chitinase with chitobiosidase activity (identified by the sequence-based approach)	45% identity to chitinase from an uncultured bacterium (Uchiyama and Watanabe, 2006) and amino acid identity to known proteins from *Chondromyces apiculatus* (41%), *Corallococcus coralloides* (40%), and *Myxococcus xanthus* (39%)	Targeted sequence-based analysis and activity-based screening of *E. coli* fosmid library	Soil, Swedish University of Agricultural Sciences, Uppsala, Sweden	Hjort et al., 2014
Six clones with antimicrobial activity: two with cell wall-degrading activity, three proteases and a lipolytic enzyme	54–31% identity to known amidase, lytic transglycosylase and proteases from *Desulfovibrio sp. U5L, Clostridium sp. CAG:1013, Myxococcus xanthus*, *Leptospira santarosai* and *Ferroglobus placidus* and to a putative lipolytic enzyme from an uncultured bacterium	Activity-based screening of broad-host cosmid shuttle vector library expressed in *Ralstonia metallidurans*	Soil, Sonoran Desert, Arizona, USA	Iqbal et al., 2014
Six clones encoding a lysostaphin gene	All six clones expressed the lysostaphin gene from the *Staphylococcus simulans* library strain	High throughput activity-based screening of *E. coli* and *Saccharomyces cerevisiae* plasmid libraries	Library derived from three native staphylococcal strains: *S. simulans*, *S. arlettae*, and *S. equorum*	Scanlon et al., 2014
**ANTI INFECTIVE**				
Two novel lactonases	One had 53% similarity to amino acid sequence from *Pseudomonas fluorescens*. The other, 57% similarity to *Nitrobacter* sp. Strain Nb-311A	Activity-based screening of *E. coli* phagemid vector, plasmid and broad-host-range vector library	Soil, University of Göttingen, Germany	Schipper et al., 2009
Clone expressing NAHL-lactonase activity	Most closely related to Zn-dependent hydrolase from *Bradyrhizobium* sp.	Functional-based screening of *E. coli* fosmid library	Pasture soil, France	Riaz et al., 2008
Two novel pairs of LuxR/LuxI genes	QS pair 1: LuxI homolog: 42% amino acid similarity to putative LuxI in *Geobacter uraniireducens* Rf4. 38% protein sequence similarity to CviI in *Chromobacterium violaceum* ATCC 31532. LuxR homolog: 33% amino acid similarity to LuxR from *Geobacter* sp. strain FRC-32 and 31% to CviR from *C. violaceum* QS pair 2: LuxI homolog: 57% similar to LuxIQS6-1 of a metagenomic clone and 40% amino acid similarity to PpuI from *Pseudomonas putida*. LuxR homolog: 37% similarity to LuxRQS10-1 in a metagenomic clone and 35% similarity to BraR in *Burkholderia kururiensis*	Activity-based screening of two fosmid libraries expressed in a biosensor *E. coli* host	Activated sludge from a coke plant, Japan. Forest soil samples, Tsukuba city, Japan	Nasuno et al., 2012
Novel bacterial NAHLase	Most likely belonging to species of unknown Proteobacterium	Activity-based screening using an *Agrobacterium tumefaciens* biosensor strain of four *E. coli* fosmid libraries	Rhizosphere of *Solanum tuberosum* that was treated with γ-caprolactone	Tannieres et al., 2013
Three novel pair of LuxR/LuxI genes	QS pair 1: 47% identity to *Nitrosospira multiformis* ATCC 25196 and 34% to *Nitrococcus mobilis* Nb-231 QS pair 2: 51% and 32% identity to *Nitrosospira multiformis* ATCC 25196 QS pair 3: both genes had 37% identity to proteins from *Sphingomonas* sp. SKA58	Activity-based screening using an *Agrobacterium tumefaciens* biosensor strain of four *E. coli* plasmid libraries	Activated sludge Soil	Hao et al., 2010
Novel NADP-dependent short-chain dehydrogenase/reductase	61% identical to chromosome segregation protein SMC in *Acidobacterium* sp. MP5ACTX8	Activity-based screening of *E. coli* phagemid vector, plasmid, and broad-host-range vector library	Soil, University of Göttingen, Germany	Bijtenhoorn et al., 2011
**ANTIBIOTIC RESISTANCE DETERMINANT**				
Novel florfenicol and chloramphenicol resistance gene	33% amino acid identity to drug resistance transporters from *Wolbachia* spp. (YP_002726856, YP_198189, and NP_966057)	Function-based screening of *E. coli* fosmid library	Soil samples from an island in the Tanana River near Fairbanks, Alaska	Lang et al., 2010
Two novel genes conferring resistance to kanamycin and ceftazidime	Both showed highest similarity to uncultured soil microorganisms	Activity-based screening of *E. coli* fosmid library	Soil from apple orchard, southern Wisconsin	Donato et al., 2010
Resistance genes to chloramphenicol, ampicillin and kanamycin. Multidrug resistant clone conferring ampicillin and kanamycin resistance	Multidrug resistant clone showed highest identity (95%) to a ß-lactamase from *Bacillus* sp. BT-192. For chloramphenicol resistance, highest homology was seen to a hypothetical protein from *Methylibium petroleiphilum*. A kanamycin resistant clone showed 55% identity to a *Microscilla* sp. protein. An ampicillin resistant clone showed 66% identity to a ß-lactamase from *Spirosoma linguale*	Functional screening of metagenomic BAC, plasmid, and phagemid vector libraries expressed in *E. coli*. Sequencing of small insert libraries.	Activated sludge	Parsley et al., 2010
Novel chloramphenicol hydrolase (resistance to chloramphenicol and florfenicol)	14 ORFs varying in similarity (30–77%) to corresponding proteins from known microorganisms. Highest similarity overall to proteins from the bacterial phylum *Proteobacteria*	Activity-based screening of *E. coli* plasmid library	Alluvial soil	Tao et al., 2012
Novel carboxylesterase	Highest identity (58%) to ß-lactamase (YP_004154831) from *Variovorax paradoxus* EPS	Activity-based screening of *E. coli* cosmid library	Soil from the Upo wetland, South Korea	Jeon et al., 2011
31 previously undescribed antibiotic resistance genes to ampicillin, amoxicillin, tetracycline, and penicillin. This includes class A and C β-lactamases and six different tetracycline resistance genes	Significant similarity to proteins from multiple genera from the ARDB and GenBank databases	Activity-based screening of *E. coli* plasmid library	Fecal samples of Herring gulsl, Appledore Island, ME and Rochester, NH, USA	Martiny et al., 2011
39 clones conferring resistance to kanamycin, gentamicin, chloramphenicol, rifampin, trimethoprim, and tetracycline	Highest homology to the following phyla: *Proteobacteria*, *Actinobacteria*, and *Firmicutes*	Activity-based screening of *E. coli* plasmid library	Urban soil, Seattle, WA, USA	McGarvey et al., 2012
110 antibiotic resistance genes conferring resistance to ß-lactams, aminoglycosides, amphenicols, sulfonamides, and tetracyclines, including 55 ß-lactamases	18 resistance genes showed 100% identity to known human pathogens	Activity-based screening of metagenomic library expressed in *E. coli* coupled with PARFuMS	11 soil samples, USA	Forsberg et al., 2012
95 unique antimicrobial resistance eDNA inserts. 10 novel β-lactamase gene families	Average of 69.5% nucleotide identity to GenBank sequences. 15 β-lactamase resistance genes showed high identity (>90%) to known human pathogens	Activity-based screening of metagenomic library expressed in *E. coli*	Human saliva and fecal samples	Sommer et al., 2009
A novel kanamycin resistance gene fusion (to a hypothetical protein domain)	N-terminus was 42% identical to AAC(6') from *Enterococcus hirae*. C-terminus was 35% identical to a hypothetical protein (CBL37632) from *Clostridiales* sp. SSC/2	Activity-based screening of *E. coli* fosmid library	Four human fecal samples	Cheng et al., 2012c
45 clones resistant to tetracycline, minocycline, aminoglycosides, streptomycin, gentamicin, kanamycin, amikacin, chloramphenicol, and rifampicin	26–92% similarity to known proteins in the GenBank database	Activity-based screening of *E. coli* plasmid library	Four agricultural soil samples, China	Su et al., 2014
Five clones conferring Fluoroquinolone resistance, cephalosporin resistance, and trimethoprim resistance	High similarity to homologs in species of *Bacillus*, *Erwinia*, *Exiguobacterium*, *Pseudomonas*	Activity-based screening of two *E. coli* plasmid libraries from cultured spinach microbiota and from uncultured spinach wash	Retail spinach	Berman and Riley, 2013
Ampicillin resistance and kanamycin resistance	Homology to *Streptococcus thermophilus* and *Lactobacillus helveticus*	Activity-based screening of an *E. coli* fosmid library	Mozzarella di Bufala Campana (MBC) Cheese, produced in Central and Southern Itlaly	Devirgiliis et al., 2014

Certain cell-to-cell communication or quorum sensing molecules and agents with quorum sensing inhibitory (QSI) activities have been also discovered through function-based screening of metagenomic libraries (Table 3). An interesting study by Nasuno et al. (2012) identified two novel sets of quorum sensing (QS) genes from the LuxI family *N*-acyl-L-homoserine lactone (AHL) synthases and their paired LuxR family transcriptional regulators. These authors constructed metagenomic libraries from an activated sludge from a coke plant and forest soil samples and functionally screened them for the presence of QS genes using a modified *E. coli* host. This biosensor strain contained a *gfp* plasmid which produced unstable GFP in response to low levels of five different AHLs, enabling the detection of QS-regulated activity. Other studies which have applied metagenomics for the exploration of QS regulation are reviewed by Kimura (2014). When it comes to treating individuals infected with, or curbing outbreaks of, antimicrobial-resistant pathogens, in some cases quorum sensing inhibitors as an anti-virulence strategy may be a useful course of action. The concept of using quorum sensing inhibitors would also be of benefit to the food industry in the control of undesirable microorganisms in food preparations or food processing environments. Schipper et al. (2009) screened a soil metagenomic library, expressed in *E. coli*, for QSI activity using an *A. tumefaciens* based bioassay. The positive clones were expressed in *Pseudomonas aeruginosa* and were found to be most likely responsible for the reduced motility and biofilm formation observed in the *P. aeruginosa* host cells expressing the proteins of interest. Of the three active clones isolated, one was found to be similar to a known lactonase, and the remaining two clones were determined to encode novel lactonases.

Certain antimicrobial strategies used in clinical settings could also be applied to the control of bacterial persistence in food development and manufacturing processes. In industrial settings contamination of food products occurs at various stages throughout the food processing cycle. The raw food itself is usually a source of initial contamination. Food can also become contaminated or re-contaminated during its processing, e.g., re-contamination of milk post-pasteurization, resulting in an unsafe or spoiled product. The removal of harmful or spoilage microorganisms from food products and the prevention of microorganisms entering or persisting in food processing is highly desirable. This needs to occur without damaging the structure, texture, taste, and overall quality of food. A potentially powerful application of functional metagenomics with respect to the food industry is screening natural sources for bioactive molecules that function as antimicrobials or inhibitory compounds for use in food safety maintenance strategies. Once the compounds have been identified and mass produced, the ultimate goal is for them to be formulated into safe sanitization products that will not influence the quality of the food product. As microorganisms are widely used and often beneficial to the food industry (e.g., cheese manufacture, brewing), the aim would be to eliminate only those microorganisms which pose a threat to food safety and quality. Screening is performed in a targeted manner to identify isolates producing compounds that inhibit or eliminate the presence of a given problematic microorganism present in the food product or processing equipment. Due to their specificity, bioactives isolated from microorganisms may be used in combination with existing sanitization products. Extremophiles are of particular interest as these could target undesirable microorganisms in extreme environments, which are often present in food processing.

Functional metagenomics can be used to combat antimicrobial resistance via two strategies; through the discovery of novel antibiotics and anti-infectives (as mentioned above) and through the identification of resistance genes in microbial populations. As resistance is transferable, horizontal gene transfer (HGT) being the most common method by which resistance is acquired by previously susceptible strains, resistant genes possessed by environmental bacteria may be acquired by human pathogens. Functional metagenomics can be used to identify novel resistance mechanisms used by bacteria in nature which may not have manifested in the clinical setting yet and so can allow one to predict possible routes *via* which resistance to current antibiotic therapies could emerge. The studies discussed below provide insight into the diversity of antimicrobial resistance mechanisms, proposing new avenues of research for tackling antibiotic resistance. They also show the value of functional metagenomics as a tool for the investigation of antimicrobial resistance, as reviewed by Mullany (2014). Donato et al. (2010) screened a metagenomic apple orchard soil library for DNA fragments that conferred antibiotic resistance to their *E. coli* host. Clones were screened for resistance to a selection of 10 antibiotics. The group reported the discovery of two novel enzymes. In one case, a metagenomic clone encoding an aminoglycoside acetyltransferase domain fused to a second acetyltransferase domain displayed resistance to kanamycin. Interestingly, sequence analysis of this clone did not predict antimicrobial resistance. The second interesting clone encoded a bifunctional protein containing a natural fusion of a ß-lactamase and a sigma factor conferring onto the host resistance to ceftazidime. Additional potential chloramphenicol resistance was predicted by sequencing this particular clone, which may evoke further analysis. Tao et al. (2012) used a TLC-based method to screen an alluvial soil-derived metagenomic library for chloramphenicol resistance. They identified a resistant clone harboring a hydrolysate which conferred to the host resistance to chloramphenicol and florfenicol, a synthetic form of chloramphenicol that was employed as a safe antibiotic treatment for use in farming. The enzyme was capable of hydrolyzing both chloramphenicol and florfenicol, with greater efficiency at hydrolyzing florfenicol. Various metagenomic studies have been carried out to identify antimicrobial resistance genes in certain foods. Antibiotic therapies for the treatment of bacterial infections in farm animals select for resistant microbes in food production chains (Hawkey, 2008). Although most microorganisms in foodstuffs are usually not pathogenic, resistant bacteria that survive on products for human consumption may transfer their resistance to opportunistic human pathogens or to the human microbiota. Certain foods (e.g., foods eaten raw) and the human gut microbiota itself may then potentially become a reservoir for antibiotic resistance genes. Retail spinach is commonly eaten raw and thus, has been linked to outbreaks of bacterial infections (Lynch et al., 2009; Wendel et al., 2009). Berman and Riley (2013) functionally screened two spinach-derived metagenomic libraries for resistance to 16 different antimicrobial agents, identifying numerous novel genes conferring resistance to ampicillin, aztreonam, ciprofloxacin, trimethoprim, and trimethoprim-sulfamethoxazole from five different active clones. Their study suggests that microorganisms in close contact with fresh food products, such as plant commensals and saprophytes, may serve as a reservoir of antimicrobial resistance genes. In a study with a similar objective, Devirgiliis et al. (2014) isolated clones displaying ampicillin and kanamycin resistance from a metagenomic library constructed from Mozzarella di Bufala Campana Italian cheese. These studies ultimately show that food products can potentially harbor bacterial species possessing clinically relevant antimicrobial resistance which may be horizontally transferred to pathogens, either directly or by an indirect route through the human microbiota.

Unusual or unexpected antimicrobial resistance mechanisms can be found in nature. Some studies investigating the resistome of uncultured bacteria have explored areas and environments which have not been previously exposed to clinical antibiotics and where endogenous microorganisms have therefore not faced selective pressure to develop antibiotic resistance. A recent study by Fouhy et al. (2014) examined the resistome of the naïve infant gut. A metagenomic library constructed from fecal samples of 22 six-month old infants who had not previously been exposed to antibiotics was screened for resistance to aminoglycoside and β-lactam antibiotics, identifying gentamicin and ampicillin resistant clones. PCR analyses were also carried out to detect DNA sequences encoding aminoglycoside and β-lactam resistance genes not successfully cloned and expressed in the library. One hundred ampicillin resistant clones were identified in their functional screen, conferring resistance *via* several β-lactamase genes. Aminoglycoside resistant clones were also identified, whose resistance was conferred by acetylation, adenylation, and phosphorylation genes. This study uncovered resistance to clinically relevant antibiotics in a naïve environment. Other studies assessing the resistome of microbial samples from remote areas where little or no antibiotic therapy has been practiced have also identified unexpected resistance (Pallecchi et al., 2008; Bartoloni et al., 2009). More recently, Clemente et al. (2015) examined the bacterial microbiome (from fecal, oral, and skin samples) of 34 Yanomami individuals from an isolated Amerindian village in South America. Among huge microbial diversity observed through 16S rRNA gene sequencing of DNA from the obtained samples, activity-based and culture-independent screening of functional and shotgun metagenomic libraries also revealed resistance genes to clinically relevant antibiotics. These studies further emphasize the diversity of the as yet uncultured microbial world by establishing that genes conveying resistance to current antibiotic therapies can be found in environments void of selective pressure.

## Conclusions and future prospects

Metagenomics grants access to the huge diversity of the microbial world and has led to significant progress among research communities and in industrial settings with respect to understanding and benefitting from unculturable microbes. Functional metagenomics is a powerful tool for the discovery of novel enzymes and bioactives sourced from as yet uncultured microorganisms. As a relatively new technology, functional metagenomics faces challenges that have yet to be overcome. However, the promise of a technique that has already proven to be fruitful even in its early years suggests that there can be significant rewards if appropriate solutions and further optimization takes place. The development of new screening and selection techniques along with faster and cheaper sequencing technologies will allow for the expansion of a very promising field in microbiology, genetics and the food and pharmaceutical industries.

This article discusses the potential of functional metagenomics to facilitate the development of novel industrial products sourced from as yet uncultured microorganisms. Nonetheless, following the identification of useful proteins and bioactives, challenges ensue in another area, that being the development of a consumer friendly and commercially viable product that can be manufactured in industrially relevant quantities, retains its activity when scaled up (for example when present in high amounts in a large industrial reaction vessel), can be purified and formulated appropriately into a finished product and maintains its stability during shipping and storage. The product also needs to be reasonably easy to use and must be applicable to current industrial demands, i.e., the product must perform efficiently under the proposed/outlined conditions to carry out the job it was bought to do. A successful reaction achieved under laboratory conditions may be difficult to reproduce on an industrial scale. Pilot plant studies must be carried out initially to identify any variables or short comings in the reaction that were not evident at the laboratory stages of development. These studies are a stepping stone between discovery of the interesting active agent and its formulation into a final commercial product. Once deficiencies and other problems have been corrected in the pilot plant phase, further studies must be conducted to qualify the agent at an industrial level and guarantee the development of a robust product that is efficient and true to its intended purpose. The acceptability of the novel enzyme or bioactive and its source microorganism to the relevant regulatory authorities must also be considered.

Once all these limitations are overcome, through access to the seemingly infinite diversity of the microbial world, functional metagenomics presents an opportunity to develop novel innovative products that offer something new and useful to industrial processes or even change for the better or make more convenient the way a current process is carried out.

### Conflict of interest statement

The authors declare that the research was conducted in the absence of any commercial or financial relationships that could be construed as a potential conflict of interest.
